# Interleukin-17 (IL-17) Expression Is Reduced during Acute Myocardial Infarction: Role on Chemokine Receptor Expression in Monocytes and Their *in Vitro* Chemotaxis towards Chemokines

**DOI:** 10.3390/toxins4121427

**Published:** 2012-11-30

**Authors:** Maria Troitskaya, Anton Baysa, Jarle Vaage, Kristin L. Sand, Azzam A. Maghazachi, Guro Valen

**Affiliations:** 1 Department of Physiology, Institute of Basic Medical Sciences, University of Oslo, Oslo N-0317, Norway; Email: maria_nemo@mail.ru (M.T.); anton.baysa@medisin.uio.no (A.B.); k.l.sand@medisin.uio.no (K.L.S.); 2 Center for Heart Failure Research, University of Oslo, Oslo N-0317, Norway; 3 Department of Emergency and Intensive Care at the Institute of Clinical Medicine, Oslo University Hospital, Oslo N-0424, Norway; Email: i.j.vaage@medisin.uio.no

**Keywords:** myocardial infarction, IL-17, monocytes, chemokines, chemokine receptors

## Abstract

The roles of immune cells and their soluble products during myocardial infarction (MI) are not completely understood. Here, we observed that the percentages of IL-17, but not IL-22, producing cells are reduced in mice splenocytes after developing MI. To correlate this finding with the functional activity of IL-17, we sought to determine its effect on monocytes. In particular, we presumed that this cytokine might affect the chemotaxis of monocytes important for cardiac inflammation and remodeling. We observed that IL-17 tends to reduce the expression of two major chemokine receptors involved in monocyte chemotaxis, namely CCR2 and CXCR4. Further analysis showed that monocytes pretreated with IL-17 have reduced *in vitro* chemotaxis towards the ligand for CCR2, *i.e.*, MCP-1/CCL2, and the ligand for CXCR4, *i.e.*, SDF-1α/CXCL12. Our results support the possibility that IL-17 may be beneficial in MI, and this could be due to its ability to inhibit the migration of monocytes.

## 1. Introduction

In acute myocardial ischemia, aseptic inflammation is induced in the heart [1]. Moreover, acute myocardial infarction is associated with systemic inflammation. Patients or experimental animals with acute infarction have increased levels of circulating cytokines, increased count of peripheral blood cells and increased *ex vivo* activation of monocytes and polymorphonuclear granulocytes [2,3]. Atherosclerosis, *per se*, is considered an autoimmune disease with activation of both innate and adaptive immunity [4,5]. The transition between atherosclerosis and acute infarction in many patients involves unstable angina, a process in which the inflammation observed during atherosclerosis is enhanced [4,6]. However, in all the three clinical conditions of atherosclerosis, unstable angina and acute infarction, it remains an open question if the systemic inflammation is a consequence of the local events in the vessel wall, a cause of the injurious process or an attempt of tissue repair upon organ injury. 

Interleukin-17 (IL-17) and interleukin-22 (IL-22) are leukocyte-derived cytokines with impact especially on epithelial cells in various tissues [7]. A broad variety of human T-cells secrete IL-17 and IL-22, as recently reviewed elsewhere [7]. While Th17 cells may possibly secrete both IL-17 and IL-22, the major source of IL-22 are Th22 cells [8]. The biological role of the cytokines is largely unknown; it is speculated that IL-17 exerts pro-inflammatory effects and is involved in the pathogenesis of several auto-inflammatory diseases, while IL-22 may have protective/regenerative effects [7]. In a recent study on 981 patients with acute myocardial infarction, low serum levels of IL-17 combined with high serum levels of the leukocyte adhesion molecule vascular cell adhesion molecule 1 (VCAM-1) was associated with a higher risk of major cardiovascular events, while patients with high IL-17 and low VCAM-1 had better outcomes [9]. In rats with induced myocardial infarction, cardiac IL-17 and target genes were upregulated post infarct, while blocking IL-17 signaling reduced apoptotic cell death post- myocardial infarct [10]. However, in neither of those studies was the major source of IL-17 production, *i.e.*, circulating Th17 cells, studied.

The purpose of the present study was to investigate the expression of IL-17 and IL-22 receptors on the cell surface of spleen cells after myocardial infarction. Furthermore, the effects of IL-17 and IL-22 on monocyte chemotaxis were investigated. 

## 2. Results

### 2.1. Percentages of IL-17-Producing Cells Are Reduced in Non-activated Splenocytes of Myocardial Infarcted Mice

**Figure 1:**
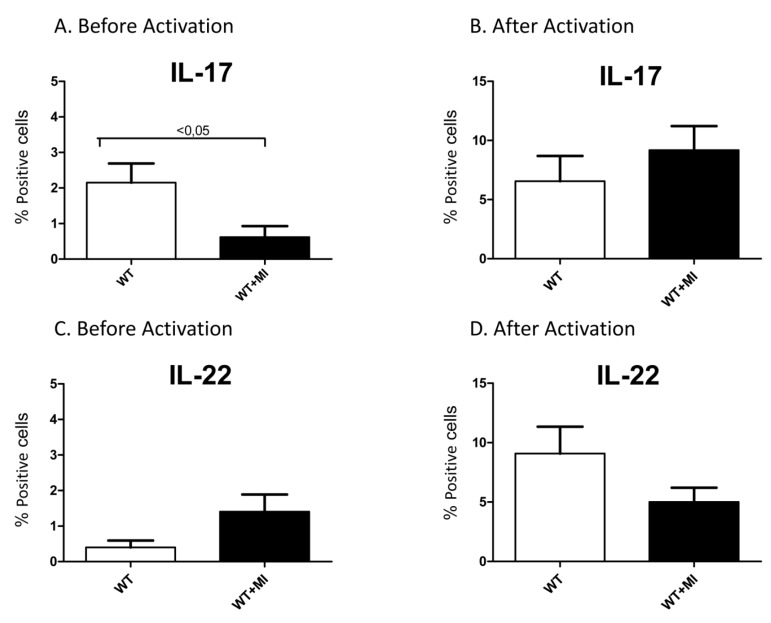
A and B. Percentages of positive splenocytes isolated from either sham-operated wild-type mice (WT) or WT with myocardial infarction induced through left coronary artery ligation three days earlier (WT + MI). Intracellular labeling with antibodies against IL-17 was performed on intact splenocytes (**A**) or splenocytes activated with PMA plus ionomycin for 4 h before labeling (**B**). C and D. Percentages of positive cells labeled intracellularly with anti-IL-22 antibodies. The cells were either unstimulated (**C**) or were activated with PMA plus ionomycin for 4 h before labeling (**D**). Mean ± SEM of 5 experiments are shown.

Spleen cells were isolated from mice subjected to permanent left coronary artery ligation three days earlier and compared with those of sham-operated mice. Intracellular staining of IL-17-producing cells show that mice suffering from myocardial infarctions (MI) had significantly less IL-17-producing cells in their spleens than non-infarcted mice (*p *< 0.05; Figure 1A). This difference disappeared upon activating splenocytes with phorbol myristate acetate “PMA” + ionomycin (Figure 1B). On the other hand, there was no statistical differences in the percentage of IL-22 producing splenocytes collected from infarcted and non-infarcted mice, regardless whether spleen cells were non-activated (Figure 1C) or activated with PMA + ionomycin (Figure 1D).

### 2.2. Immune Cells Express IL-17 Receptors and Not IL-22 Receptors

**Figure 2:**
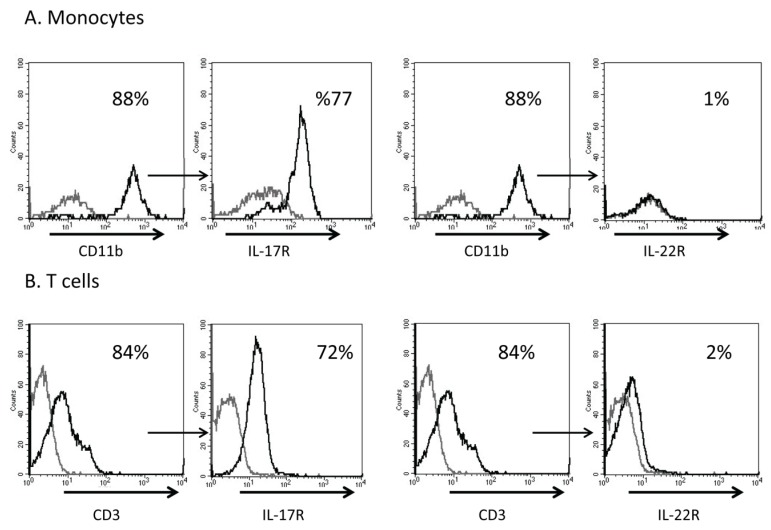
(**A**) Monocytes were isolated by monocyte enrichment kit and were labeled with anti-CD11b. About 88% of monocytes were labeled with this antibody. This population was then stained with either anti-IL-17R or anti-IL-22R; (**B**) T cells were isolated using a T cell enrichment kit, labeled with anti-CD3 and then with anti-IL-17R or anti-IL-22R. One representative experiment indicating the percentages of positive cells is shown.

Because IL-17, and possibly IL-22, secreted from inflammatory cells might affect cells of the immune system, we examined the expression of IL-17R and IL-22R on T cells and monocytes, cells presumably activated during myocardial infarction. About 80% of monocytes isolated based on the expression of CD11b also expressed IL-17R, but not IL-22R (Figure 2A). Similarly, T cells, which were isolated based on the expression of CD3, also expressed IL-17R, but not the IL-22R (Figure 2B). 

### 2.3. Effect of IL-17 on Chemokine Receptor Expression

**Figure 3:**
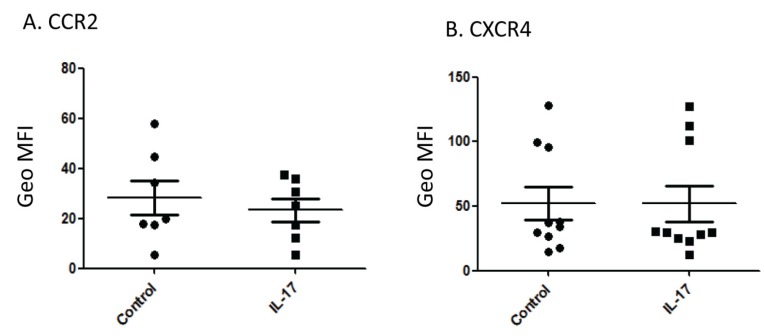
IL-17 may reduce the expression of CCR2 and CXCR4 on the surface of monocytes. Monocytes (1 × 10^6^) were either left intact (Control) or were pretreated with 250 ng/mL of IL-17 overnight*. *They were washed and then examined for the expression of CCR2 (**A**) or CXCR4 (**B**). The geometric mean of fluorescence intensity (Geo MFI) is shown. Each dot represents one independent experiment.

As monocytes expressed the IL-17R, and not the IL-22R, we pursued investigating the effect of IL-17 on these cells, which are known to transmigrate into ischemic myocardial tissue after infarction. First we investigated whether IL-17 affects the expression of chemokine receptors on monocytes. From the chemokine receptors examined (CCR2, CCR5, CCR6, CCR7, CXCR3 and CXCR4), we noticed a tendency towards reduced expression of CCR2 and CXCR4 on the surface of monocytes, although this was not statistically significant (Figure 3A,B).

**Figure 4:**
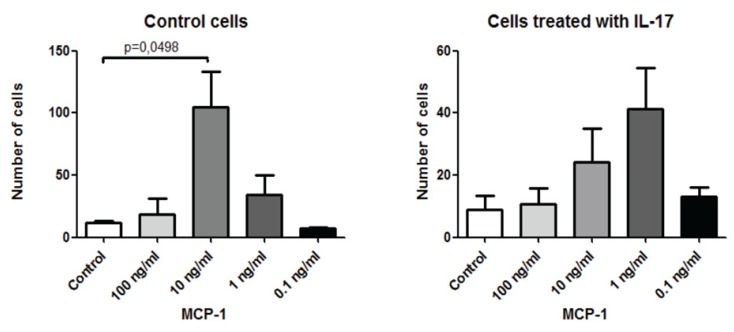
IL-17 inhibits monocytes chemotaxis towards MCP-1/CCL2. Monocytes were either untreated (control cells) or pretreated with 250 ng/mL of murine recombinant IL-17 overnight. The cells were washed, labeled with calcein-AM and then examined for migration towards different concentrations of MCP-1/CCL2. Two hours later, calcein-AM-labeled cells were collected and counted in the flow cytometer. Mean ± SEM of two experiments are shown.

### 2.4. IL-17 Affects the* in Vitro* Chemotaxis of Monocytes towards MCP-1/CCL2

Based on the above findings, we sought to examine the effect of this cytokine on chemokine-induced monocyte chemotaxis. For this, we used the ligands for CCR2 and CXCR4, namely MCP-1/CCL2 and SDFα-1/CXCL12, respectively. Results in Figure 4A demonstrate that 10 ng/mL of MCP-1/CCL2 significantly induced the chemotaxis of monocytes as compared to the control, where media was used instead of the chemokine (*p *< 0.05; Figure 4A). This increase in chemotaxis disappeared upon pretreatment with IL-17 (Figure 4B). 

### 2.5. IL-17 Affects the *in Vitro* Chemotaxis of Monocytes towards SDF-1α/CXCL12

**Figure 5:**
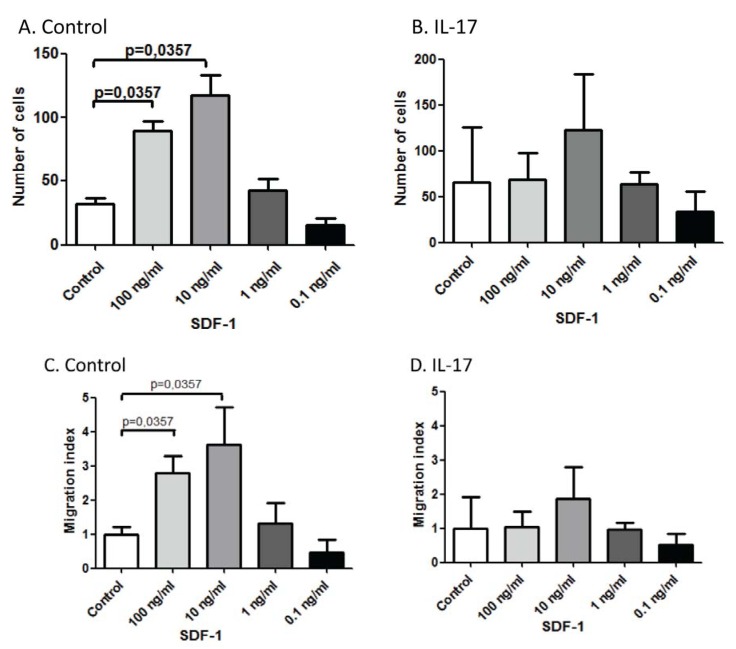
IL-17 inhibits the chemotaxis of monocytes towards SDF-1α/CXCL12. (**A**) and (**B**) Monocytes were either untreated (control cells) or pretreated with 250 ng/mL IL-17 overnight. The cells were washed, labeled with calcein-AM and then examined for migration towards various concentrations of SDF-1α/CXCL12; (**C**) and (**D**) Migration index was calculated by measuring the numbers of migrating cells into the lower filters of the chemotaxis chambers. Mean ± SEM of two experiments are shown.

Next, we examined the effect of SDF-1α/CXCL12, which binds CXCR4, and observed that 10 and 100 ng/mL concentrations of this chemokine significantly induced the chemotaxis of monocytes (*p* < 0.04; Figure 5A). Similar to its effect on MCP-1/CCL2-induced chemotaxis, IL-17 pretreatment abolished the chemotaxis induced by SDF-1α/CXCL12 (Figure 5B). Chemotaxis towards SDF-1α/CXCL12 was repeated by measuring the migration index rather than counting the numbers of calcein-AM-labeled cells into the lower wells. Exactly similar results were observed, *i.e.*, 10 and 100 ng/mL of CXCL12 induced the chemotaxis of monocytes into the lower wells of the chemotaxis chamber (Figure 5C), and this effect disappeared upon pretreating these cells with IL-17 (Figure 5D). 

## 3. Discussion

The roles of immune cells and their soluble factors in atherosclerosis and acute myocardial infarction (MI) are not clearly defined. Here we report that the percentages of IL-17, but not IL-22, producing cells are reduced in spleen cells of mice with MI. IL-17 is released from Th17 cells [11] or NK17/NK1 cells [12], whereas the related cytokine IL-22 is released from either Th-22 [8] or NK-22 cells [13]. IL-17 is considered an inflammatory molecule, as it induces the production of IL-8/CXCL8 [14], which recruits neutrophils. IL-17 also stimulates the production of matrix metalloproteinase-2, 3, 9 and 13 [15], and facilitates the proliferation of endothelial cells [16]. Consequently, IL-17 or cells that secrete it, such as Th17, are considered culprits in autoimmune diseases, including multiple sclerosis [17], rheumatoid arthritis [18], psoriasis [19] and asthma [20]. However, the role of IL-17 in MI is controversial. Barry *et al.* [10] reported that IL-17 and its receptors are increased during MI in rats. This study does not contradict the present findings, as we measured splenocytes for IL-17 expression three days post MI in mice, while they measured gene and protein expression of IL-17 in left ventricles of rats 24 h after MI induction. IL-17 may be involved in reducing inflammation after ischemia-reperfusion injury to the kidney, evident by modified infiltration of neutrophil granulocytes in acutely injured kidneys of mice deficient of IL-17 [21]. Mice treated with the bacterium *Porphyromonas gingivalis* had induced myocarditis and/or MI, accompanied by increased levels of IL-17 [22]. IL-17 knockout mice had reduced infiltration of monocytes and neutrophil granulocytes in myocardial tissue, suggesting that IL-17 may play an important role after injury. Further, IL-17 induced the expression of CXCL1 mRNA levels, which may recruit neutrophils into the myocardium [10,21]. In order to understand whether IL-17 may influence inflammation related to MI, we sought to investigate whether IL-17 might affect the recruitment of monocytes, cells that are involved in atherosclerosis and MI [23]. We observed that IL-17 tends to reduce the expression of CCR2 and CXCR4 on the surface of monocytes. To corroborate this finding with the recruitment of monocytes, we performed *in vitro* chemotaxis assay and observed that pretreatment of monocytes with IL-17 results in reduced chemotaxis. In particular, we observed that IL-17-pretreated monocytes have reduced chemotaxis towards the ligands for CCR2, *i.e.*, MCP-1/CCL2, and CXCR4, *i.e.*, SDF-1α/CXCL12. Both these chemokines are involved in MI. 

The administration of CXCR4 antagonist AMD3100 resulted in reduced scar formation, corroborated with improved cardiac contractility after MI [24]. Further, deficiency of CXCR4 limits infarct size, which could be due to reduced infiltration of monocytes after development of MI [25]. In the present study, we used spleen cells as a model of circulating inflammatory cells, as in mice the cells in the spleen are representative of those in the circulation. We speculate that our observations may be relevant to the known recruitment of monocytes from the circulation into infarcted myocardium *in vivo*. It is possible that cardiac outcome after MI may be influenced by the release of IL-17, which may limit the recruitment of inflammatory monocytes into sites of injury. This speculation and the present results are supported by the findings of Taleb *et al.* [26], who reported that IL-17 down-regulates the expression of VCAM on mouse endothelial cells. The same authors reported that IL-17 inhibits the adherence of mononuclear cells to pre-activated human umbilical vein endothelial cells [9]. Hence, IL-17 should not only be looked at as an inflammatory molecule that damages the injured tissues. This is in line with another study showing that IL-17 may be beneficial for inflammatory colitis disease [27]. In this study, it was observed that Th17 cells inhibit the development of Th1 cells and, consequently, the release of IFN-γ. Hence, in the absence of Th17/IL-17, Th1 cells induce strong colitis disease. Finally, the observation that patients with higher IL-17 levels had reduced risk of major cardiovascular events [9] may provide further evidence of a beneficial role of IL-17.

## 4. Experimental Section

### 4.1. Animals

Male C57Bl/6 mice 24–28 days old (NOVA-SCB, Nittedal, Norway) were used in this study. All animals were allowed at least five-to-seven days of acclimatization after shipment to the animal stable before the actual experiments. The mice had conventional microbial status and were kept under regulated temperature 22–23 °C and relative humidity 55% ± 5%, with an alternating light: dark cycle (12:12). Animals had free access to water and chow. The experiments were approved by the Norwegian Animal Health Authority and were performed under the principles of laboratory animal care (Guide for the Care and Use of Laboratory Animals published by the United States National Institute of Health, NIH Publication no. 85-23, revised 1996).

### 4.2. Antibodies

PE-conjugated monoclonal rat anti-mouse CCR2, PE-conjugated monoclonal rat anti-mouse CCR6, PE-conjugated monoclonal rat anti-mouse CCR7, PE-conjugated monoclonal rat anti-mouse CXCR3, PE-conjugated monoclonal rat anti-mouse CXCR4, PE-conjugated monoclonal rat anti-mouse IL-22Rα, CXCR4 PE-conjugated IgG2A isotype control and PE-conjugated IgG2B isotype control were purchased from R&D Systems (R&D Systems Europe Ltd., Abingdon, UK). PE-conjugated monoclonal rat anti-mouse CCR5 (CD 195) and PE-conjugated IgG2c isotype control were purchased from BD Biosciences. PE-conjugated monoclonal rat anti-mouse IL-17RA (CD 217) was purchased from eBioscience. FITC-conjugated monoclonal rat anti-mouse CD 11b, FITC-conjugated monoclonal rat anti-mouse CD 14 and FITC-conjugated kappa monoclonal mouse IgG2A were purchased from Abcam (Cambridgeshire, UK). FITC-conjugated monoclonal rat anti-mouse CD3 was purchased from Termo Scientific.

### 4.3. Coronary Artery Ligation

Mice were anesthetized with isofluran and intubated with the maintenance of isofluran 1.5% mixed with pure oxygen. Mechanical ventilation (Model 874 092, B. Braun) was used to keep a respiratory rate of 133 per min. For induction of myocardial infarction, *in vivo* permanent occlusion of the left anterior descending coronary artery was performed after a left-sided thoracotomy, as previously described in detail [28]. The body temperature was kept at 37 °C during surgery with a heating plate. After ligation, the thoracic cavity was closed and skin and muscle was reattached. For the sham operation, all these steps were performed with the exception of coronary artery ligation. Sodium saline (0.5 mL) and Temgesic (0.1 mg/kg) were administered subcutaneously for rehydration and analgesia after the operation. Mice were extubated when spontaneous respiration occurred and kept in a “mini intensive care unit” with 100% humidity and 30 °C during the first postoperative day. Temgesic was administered postoperatively by the veterinarian any time pain-associated behavior of the mice was observed.

In the first series of experiments, wild-type C57BL/6 mice were subjected to either *in vivo *coronary artery occlusion (*n* = 5) or sham operation (*n* = 5). Spleens were harvested for cell isolation three days later from isofluran anesthetized mice and compared with spleens from sham operated mice. In subsequent experiments, spleens were harvested from C57Bl/6 after cervical dislocation for the end-points of flow cytometry analysis or chemotaxis and migration, as described below. 

### 4.4. Preparation of Spleen Cells

Spleen cells harvested from mice with coronary artery ligation or sham-operation were subjected to flow cytometry analysis after stimulation with phorbol myristate acetate (PMA, 20 ng/mL) combined with ionomycin (2 µM) for 22–24 h, and compared with non-treated cells collected the same day as the spleens were sampled. Spleens were extracted, placed in culture medium and homogenized under sterile conditions. The culture medium consisted of RPMI-1640 supplemented with 10% FCS, 100 U/mL penicillin, 100 µg/mL streptomycin, 2 mM L-glutamine, 1% nonessential amino acids (Gibco BRL Life Technologies, the Netherlands) and 50 µM β-mercaptoethanol (Sigma-Aldrich, Oslo, Norway). After histopaque (Sigma-Aldrich, Oslo, Norway) separation, the monocyte population was purified using either adherence or a Mouse Monocyte Enrichment negative selection kit (StemCell Technologies SARL, Grenoble, France). T-cells were positively selected using a Monocyte Enrichment negative selection kit (StemCell Technologies SARL, Grenoble, France). After isolation, cells (1 × 10^6^/mL) were divided in two groups, incubated with culture medium and with or without recombinant murine IL-17A (250 ng/mL, PeproTech) overnight at 37 °C in a 5% CO_2_ incubator. 

### 4.5. Flow Cytometry Analysis

Cells incubated with or without IL-17 were resuspended in PBS containing 0.1% sodium azide and labeled with 1 µg/mL PE-conjugated monoclonal rat anti-mouse CCR2, CCR5, CCR6, CCR7, CXCR3, CXCR4 (R&D Systems Europe Ltd., Abingdon, UK), IL-17RA (eBioscience, San Diego, CA, USA), IL-22Rα (R&D Systems) or FITC-conjugated monoclonal rat anti-mouse CD3, CD11b and CD14 for 45 min at 4 °C. As a control, cells were incubated with isotype control antibodies. The cells were washed twice and examined in flow cytometer (FACSCalibur, Becton Dickinson Biosciences, San Jose, CA, USA). Gating was performed according to the isotype control. To investigate if the spleen cells express IL-17 or IL-22 receptors (IL-17R and IL-22R), spleen cells were incubated with antibodies against CD3 (T-cell marker), CD-11b (monocyte marker) together with antibodies against IL-17R and IL-22R and sorted by flow cytometric analysis. As spleen cells contained the receptor for IL-17, but not IL-22, spleen cells were isolated and incubated with recombinant murine 250 ng/mL IL-17A (PeproTech EC Ltd., England) over night, compared with unstimulated cells, and sorted by FACS to evaluate the expression of CCR2, CCR4, CCR5,CCR6, CCR7, CXCR3 and CXCR4.

### 4.6. *In Vitro* Chemotaxis Assay

To evaluate a possible role of IL-17 on chemotaxis, monocytes were incubated in the presence or absence of 250 ng/mL recombinant murine IL-17 overnight. Chemotaxis assay was done using Nuclepore blind well chemotaxis chambers with a lower well volume of 200 µL used. A maximum volume of 200 µL medium containing RPMI plus 0.1% BSA was placed in the lower wells in the presence or absence of 0.1–100 ng/mL of MCP-1/CCL2 or SDF-1α/CXCL12 chemokines (PeproTech, England). Cells (5 × 10^5^) were placed in the upper compartments and incubated for three hours at 37 °C in a 5% CO_2_ incubator. The filters were removed, dehydrated, cells were fixed with methanol, stained with 15% modified Giemsa for 10 min and then mounted on glass slides. Cells in 10 high-power fields were counted and averaged for each sample. Migration index (MI) was calculated as the number of cells migrating toward the concentration gradients of chemokines divided by the number of cells migrating toward medium only. In the second protocol, cells (1 × 10^6^/mL) were incubated with 10 µg Calcein-AM and incubated for 45 min at 37 °C in 24 well plates. The cells were spun down at 500 rpm for 5 min, washed and collected. Calcein-AM labeled cells (5 × 10^5^) were incubated in the chemotaxis chambers, as described above for 3 h. After this time, cell suspensions were collected from the lower wells and fluorescence intensity of the calcein AM-loaded cells was measured in a BioTek FLX TBI plate reader using 485/528 nm fluorescence filters.

### 4.7. Statistical Analysis

A *t*-test was used when data were evenly distributed, while a Mann-Whitney test was used for data with a non-gaussian distribution. *p *< 0.05 was considered significant.

## 5. Conclusions

We provide novel evidence showing that the inflammatory cytokine IL-17 may have beneficial effects in mice with myocardial infarction. This molecule inhibits the *in vitro* chemotaxis of monocytes towards MCP-1/CCL2 and SDF-1α/CXCL12. This activity may be related to the IL-17 effect on the expression of CCR2 and CXCR4 on the cell surface of these monocytes, although other mechanisms might also be important, such as modulating the affinity of the receptors for their chemokine ligands. Consequently, IL-17 may potentially be used therapeutically for treating MI, considering that it may ameliorate, rather than exacerbate, the disease. 
